# Supplementary material for: From sequence to enzyme mechanism using multi-label machine learning
Source: BMC Bioinformatics. 2014 May 19;15:150. doi: 10.1186/1471-2105-15-150 (PMC4229970; doi:10.1186/1471-2105-15-150)
Supplement: Additional file 2 — Java code of ml2db. Additional file ml2db_code.tar.gz contains the Java source code to run the multi-label machine learning experiments and save the results to database. The code’s Javadoc is included. [file 1471-2105-15-150-S2.zip › additional file 2/ml2db/ml2db/doc/main/ml2db/mulan/learn/traintest/TrainTestEvaluatorSerialised.html]

TrainTestEvaluatorSerialised


JavaScript is disabled on your browser.


- Overview
- Package
- Class
- Use
- Tree
- Deprecated
- Index
- Help

- Prev Class
- Next Class

- Frames
- No Frames

- All Classes

- Summary:
- Nested |
- Field |
- Constr |
- Method

- Detail:
- Field |
- Constr |
- Method


main.ml2db.mulan.learn.traintest

## Class TrainTestEvaluatorSerialised

- java.lang.Object
- - mulan.evaluation.Evaluator
  - - main.ml2db.mulan.learn.evaluation.EvaluatorNoAUC
    - - main.ml2db.mulan.learn.crosseval.CrossEvaluator
      - - main.ml2db.mulan.learn.traintest.TrainTestEvaluator
        - - main.ml2db.mulan.learn.traintest.TrainTestEvaluatorSerialised

- ---

    

  ```
  public class TrainTestEvaluatorSerialised
  extends TrainTestEvaluator
  ```

  Class

  Version:
  :   21 Oct 2010

  Author:
  :   Luna De Ferrari ldf(at)st-andrews.ac.uk

- - ### Constructor Summary

    Constructors

    Constructor and Description

    `TrainTestEvaluatorSerialised(TrainTestExperimenter mulanTrainTestExperimenter,
    java.lang.String serialisedTrainedModel)`
  - ### Method Summary

    Methods

    | Modifier and Type | Method and Description |
    | --- | --- |
    | `java.lang.String` | `getTrainedModelFile()` |

    - ### Methods inherited from class main.ml2db.mulan.learn.traintest.TrainTestEvaluator

      `getExperimenter, getPredSaver, trainTestValidate`
    - ### Methods inherited from class main.ml2db.mulan.learn.crosseval.CrossEvaluator

      `evaluate, internallyCrossValidate`
    - ### Methods inherited from class main.ml2db.mulan.learn.evaluation.EvaluatorNoAUC

      `evaluate, evaluate, getEndTime, getStartTime, getStrictness, isStrict, setEndTime`
    - ### Methods inherited from class mulan.evaluation.Evaluator

      `checkData, checkLearner, crossValidate, crossValidate, evaluate, evaluate, setSeed`
    - ### Methods inherited from class java.lang.Object

      `equals, getClass, hashCode, notify, notifyAll, toString, wait, wait, wait`

- - ### Constructor Detail


    - #### TrainTestEvaluatorSerialised

      ```
      public TrainTestEvaluatorSerialised(TrainTestExperimenter mulanTrainTestExperimenter,
                                  java.lang.String serialisedTrainedModel)
      ```
  - ### Method Detail


    - #### getTrainedModelFile

      ```
      public java.lang.String getTrainedModelFile()
      ```


- Overview
- Package
- Class
- Use
- Tree
- Deprecated
- Index
- Help

- Prev Class
- Next Class

- Frames
- No Frames

- All Classes

- Summary:
- Nested |
- Field |
- Constr |
- Method

- Detail:
- Field |
- Constr |
- Method
